# Flavoprotein-Mediated Tellurite Reduction: Structural Basis and Applications to the Synthesis of Tellurium-Containing Nanostructures

**DOI:** 10.3389/fmicb.2016.01160

**Published:** 2016-07-26

**Authors:** Mauricio Arenas-Salinas, Joaquín I. Vargas-Pérez, Wladimir Morales, Camilo Pinto, Pablo Muñoz-Díaz, Fabián A. Cornejo, Benoit Pugin, Juan M. Sandoval, Waldo A. Díaz-Vásquez, Claudia Muñoz-Villagrán, Fernanda Rodríguez-Rojas, Eduardo H. Morales, Claudio C. Vásquez, Felipe A. Arenas

**Affiliations:** ^1^Centro de Bioinformática y Simulación Molecular, Universidad de TalcaTalca, Chile; ^2^Departamento de Biología, Facultad de Química y Biología, Universidad de Santiago de ChileSantiago, Chile; ^3^Facultad de Ciencias de la Salud e Instituto de Etnofarmacología, Universidad Arturo PratIquique, Chile; ^4^Facultad de Ciencias de la Salud, Universidad San SebastiánSantiago, Chile

**Keywords:** tellurite, tellurite reduction, flavoprotein, tellurite nanostructures, *Escherichia coli*

## Abstract

The tellurium oxyanion tellurite (TeO_3_^2-^) is extremely harmful for most organisms. It has been suggested that a potential bacterial tellurite resistance mechanism would consist of an enzymatic, NAD(P)H-dependent, reduction to the less toxic form elemental tellurium (Te^0^). To date, a number of enzymes such as catalase, type II NADH dehydrogenase and terminal oxidases from the electron transport chain, nitrate reductases, and dihydrolipoamide dehydrogenase (E3), among others, have been shown to display tellurite-reducing activity. This activity is generically referred to as tellurite reductase (TR). Bioinformatic data resting on some of the abovementioned enzymes enabled the identification of common structures involved in tellurite reduction including vicinal catalytic cysteine residues and the FAD/NAD(P)^+^-binding domain, which is characteristic of some flavoproteins. Along this line, thioredoxin reductase (TrxB), alkyl hydroperoxide reductase (AhpF), glutathione reductase (GorA), mercuric reductase (MerA), NADH: flavorubredoxin reductase (NorW), dihydrolipoamide dehydrogenase, and the putative oxidoreductase YkgC from *Escherichia coli* or environmental bacteria were purified and assessed for TR activity. All of them displayed *in vitro* TR activity at the expense of NADH or NADPH oxidation. In general, optimal reducing conditions occurred around pH 9–10 and 37°C. Enzymes exhibiting strong TR activity produced Te-containing nanostructures (TeNS). While GorA and AhpF generated TeNS of 75 nm average diameter, E3 and YkgC produced larger structures (>100 nm). Electron-dense structures were observed in cells over-expressing genes encoding TrxB, GorA, and YkgC.

## Introduction

Interest in some particular metal(loid)s has grown considerably in recent years because of their increasing applicability in the chemical, metallurgy, optical, and medical industry. For instance, germanium in combination with tellurium, antimony, and/or bismuth allows the production of optical devices such as DVD-RAM and DVD-RW. There is also a growing demand from nanotechnology, where Te-based nanostructures (TeNS) are used in the production of solar energy devices and in biomedicine (Bao et al., 2010). Tellurium and other elements such as Hg, Pb, and Mo, among others, are commonly obtained as byproducts of copper, nickel, silver, or gold refining. Their accumulation in the metal-refining process has resulted in increased environmental pollution, which has become a worldwide concern (Turner, 2001; Dittmer, 2003).

It is therefore of great ecological and scientific interest to diminish the amount of this kind of toxicants as well as to clean up metal-polluted environments. The increasing number of communications dealing with the isolation of bacteria naturally resistant to metals from clinical (Bradley, 1985; Taylor, 1999) and environmental samples (Summers and Jacoby, 1977; Amoozegar et al., 2008) reflects an indirect evidence of such pollution.

Metal(loid)-bacteria interactions play a critical role in a number of biotechnological applications including bioleaching, biomineralization, and bioremediation (Mandal et al., 2006). Microbial systems are good candidates for decontaminating sites polluted with soluble metal ions either by reducing and/or precipitating them to less toxic, nanoclustered insoluble forms (Klaus-Joerger et al., 2001; Konishi et al., 2007; Suresh, 2012). Although studied for a long time, the molecular basis of bacterial metal reduction is yet to be fully elucidated. In fact, only partial progress has been made in deciphering bacterial metal-reducing ability as well as identifying novel microorganisms involved in these processes.

Metal(loid) reduction generally leads to the formation of nanoparticles or nanostructures that possess unique properties for applications in nanotechnology. This field is in rapid expansion by creating new functional materials, devices and systems within a nanometer scale (Schmid, 2004). However, most of the existing chemical synthesis procedures require high temperature, anaerobic conditions (to prevent oxidation of reagents) or the presence of a number of toxic components that ultimately limit their general applications. Furthermore, it is not easy to control the size, shape, and properties of the synthesized nanoparticles (Turner et al., 2012). For these reasons, using microbiological methods for nanostructure (NS) production is considered a safe, economically, and environmentally friendly process. However, the nanotechnological breakthrough to utilize microorganisms for precipitating nanoclusters of various metal(loid)s is still in early stages (Tsezos, 2007; Suresh, 2012).

Regarding tellurium, its abundance in the Earth’s crust is very low (0.027 ppm average; Turner et al., 2012), and it is most often found in copper- and sulfur-bearing ores or associated with other metals such as gold and bismuth. Because of its metal-like characteristics, tellurium can exist in various redox states: telluride [Te(II), Te^2-^], elemental tellurium [Te(0), Te^0^], tellurite [Te(IV), TeO_3_^2-^], and tellurate [Te(VI), TeO_4_^2-^]. Although Te^0^ seems to display no toxicity, tellurite is extremely noxious to most bacteria even at concentrations as low as 1 μg ml^-1^ (Taylor, 1999; Chasteen et al., 2009). This is even more dramatic when compared with the toxicity of other metal(loid)s of environmental concern such as chromium, iron, cadmium, and copper, among others, which become toxic at concentrations ∼100-fold higher (Nies, 1999). Although not much is known about tellurite toxicity for humans, its bactericidal activity was recognized prior to the antibiotic era (Fleming, 1932).

It has been shown that reduced cellular thiols (RSH), especially glutathione (GSH), represent tellurite targets that are oxidized in the presence of the toxicant (Turner et al., 2001). Upon TeO_3_^2-^ exposure, various bacteria such as *Escherichia coli* (Pérez et al., 2007), *Pseudomonas pseudoalcaligenes* (Tremaroli et al., 2007) and *Rhodobacter capsulatus* (Borsetti et al., 2005) develop an oxidative stress status exhibiting increased concentrations of reactive oxygen species (ROS), especially superoxide, which in turn affects a number of macromolecules and/or metabolic pathways (Imlay, 2003, 2008).

Metal(loid) resistance mechanisms commonly found in bacteria, eukaryotes, and archaea include adsorption, volatilization, releasing chelating compounds, eﬄux pumps, decreased toxicant influx, enzymatic detoxification, and intracellular sequestration (Nies, 1999; Sochor et al., 2011; Srivastava et al., 2013). However, there is no single strategy providing a universal resistance mechanism to all toxic metals, probably reflecting their distinctive physicochemical properties.

In the particular case of tellurite, bacterial resistance is a phenomenon that does not seem to be related to specific gene products and rather represents a multifactor response. Although tellurite sensitive bacteria also reduce the toxicant when growing at sub lethal concentrations, there is growing evidence that its reduction greatly mitigates the deleterious effects that it causes in the cell.

Enzyme-mediated tellurite reduction has been documented for nitrate reductases (Avazéri et al., 1997; Sabaty et al., 2001), terminal oxidases from the respiratory chain of diverse Gram-negative bacteria (Trutko et al., 2000; Díaz et al., 2014), catalase (Calderón et al., 2006), isocitrate dehydrogenase and 6-phosphogluconate dehydrogenase from *E. coli* (Reinoso et al., 2012; Sandoval et al., 2015), glutathione reductase (GorA) from *Pseudomonas* sp. BNF22 (Pugin et al., 2014), and dihydrolipoamide dehydrogenase (Castro et al., 2008, 2009; Arenas et al., 2014a). Since no common structural patterns are evident among these enzymes, the aim of this work was to look for common protein motifs in these tellurite-reducing proteins. Results from bioinformatic analyses led to the prediction of putative enzymes with the ability to transform tellurite, which were then characterized. It is expected that this work will lead to a better understanding of the basic principles of tellurite reduction and tellurium-containing nanostructures synthesis.

## Materials and Methods

### Growth Conditions

Bacteria were routinely grown in LB medium (Sambrook and Russell, 2001) with shaking at 37°C. Growth was started by inoculating fresh LB medium with 1:100 dilutions of overnight cultures. For growth in solid medium, LB-agar (2%) plates were incubated overnight at the same temperature. When required, cells were grown in the presence of chloramphenicol (25 μg ml^-1^) or ampicillin (100 μg ml^-1^; **Table 1**). Expression of cloned genes (ASKA collection) was induced with 1 mM IPTG for 6–10 h.

**Table 1:** Kinetic parameters and optimal conditions for TR activity of the indicated *E. coli* flavoenzymes.

Enzyme	TR activity (U/mg protein)	*K*_m_ apparent (mM)	*K*_i_ apparent (mM)	*V*_max_ (U/mg protein)	pH	Temp (°C)	Cofactor
GorA	32,228.6 ± 588.8	0.08947	0.2327	6,314	9	37	NADPH
E3	2,788.1 ± 148.9	0.04794	0.06301	84.5	6	37	NADPH
TrxB	1,423.9 ± 181.7	0.1145	0.03713	9,586	10	37	NADPH
AhpF	931.1 ± 17.6	0.8196	–	77,875	10	37	NADH
YkgC	874.7 ± 41.8	0.5171	–	2,696	9	37	NADH
NorW	664.1 ± 231.1	0.6949	–	5,347	11	42	NADH

*TR activity and kinetic parameters were determined as described in Section “Materials and Methods.*

### Bioinformatic Analysis

#### Sequence Alignments and Motif Search

Amino acid sequences of enzymes predicted to display TR activity were obtained from UNIPROT database (Li et al., 2001). Direct comparison was based on multiple sequence alignments using the CLUSTALW (Thompson et al., 1994) and MAFFT (Katoh et al., 2002) software packages. Each protein sequence was analyzed through several biological databases to find common characteristics (InterPro, PROSITE, P-fam, CATH, SCOP database).

#### Structure Comparisons

Structural comparisons were carried out through alignments using the STAMP (Russell and Barton, 1992) and SSAP methods (Orengo and Taylor, 1996) with the following *E. coli* proteins available in PDB databases: E3 (*lpdA*) component of the pyruvate dehydrogenase complex (PDB_ID: 4JDR, Chandrasekhar et al., 2013), GorA (PDB_ID: 1GER, Mittl and Schulz, 1994), alkyl hydroperoxide reductase (AhpF; PDB_ID: 1FL2, Bieger and Essen, 2001) and thioredoxin reductase (TrxB; PDB_ID: 1CL0, Lennon et al., 1999).

Important distances were measured between the most relevant atoms for enzyme activity using the VMD software (Humphrey et al., 1996). Distances between Cα-Cα relative to disulfide redox (distance 1), from the SH group of the first (distance 2) and second (distance 3) cysteine of the disulfide bridge and C4 of the FAD’s isoalloxazine ring were determined. Distance 4 was calculated from the SH groups from the redox active site (Cys1: SH – Cys2: SH) and distance 5 was calculated from the SH group of the second cysteine of the disulfide bridge (Cys2) and N5 nitrogen from the FAD’s isoalloxazine ring.

#### Molecular Model of YkgC from *E. coli*

*Escherichia coli* YkgC shares 30% amino acid sequence identity with *P. aeruginosa* mercuric reductase (MerA; PDB_ID: 1ZK7; Ledwidge et al., 2005) and this was used as a template to build a homology model using the Modeller software (Sali and Blundell, 1993). The model was validated using Anolea (Melo et al., 1997) and optimized through energy minimization (5,000 steps) and molecular dynamics during 4 ns using the NAMD program (Phillips et al., 2005). Simulation conditions were as described previously (Arenas et al., 2014a).

### Cloning Flavoprotein Genes from the Tellurite-Resistant Environmental Strain BNF01 and from pTP6 Plasmid

*Staphylococcus haemolyticus* BNF01 (Arenas et al., 2014b) *ahpF* and *trxB* genes were amplified using specific primers (Supplementary Table S1). Since *E. coli* lacks the MerA gene and given that bioinformatic data suggested that MerA could display TR activity, the *merA* gene was amplified from the environmental plasmid pTP6^1^. PCR products were individually inserted into the vector Champion^TM^ pET101 Directional TOPO Expression (Invitrogen^®^) to generate plasmids pET/*ahpF*, pET/*trxB*, and pET/*merA*. Correct insertion of genes was checked by PCR using specific primers (Supplementary Table S1). Their identity was confirmed by DNA sequencing.

### Protein Purification

*Escherichia coli* flavoproteins were purified using cells from the ASKA collection (Kitagawa et al., 2005). *S. haemolyticus* BNF01 genes encoding the selected flavoproteins and *merA* from plasmid pTP6 were cloned into the pET101/D-TOPO vector (Invitrogen^®^) and transformed into *E. coli* BL21 (DE3). Cells were grown at 37°C to OD_600_ ∼ 0.6 and induced with 1 mM IPTG for 6–10 h with vigorous shaking. Cells were suspended in binding buffer (20 mM sodium phosphate, pH 7.4, 0.5 M NaCl, 20 mM imidazole), supplemented with 0.1 mM PMSF and disrupted by sonication. The cell debris was discarded by centrifugation at 14,000 × *g* for 15 min at 4°C and His-tagged proteins present in the crude extracts were purified by affinity chromatography columns (HisTrap HP, GE Healthcare^®^). After extensive washing with binding buffer, bound proteins were eluted with elution buffer (same as binding buffer but containing 0.5 M imidazole). Proteins were dialyzed against 50 mM Tris-HCl buffer pH 7.4 for 2 h. Protein concentration was determined as described earlier (Bradford, 1976), and SDS-PAGE was carried out to assess enzyme purity.

### Enzyme Activity and Biochemical Characterization

Tellurite reductase activity was determined in a final volume of 200 μl of 50 mM Tris-HCl buffer pH 7.4, 0.15 mM K_2_TeO_3_, 1 mM NAD(P)H, 1 mM β-mercaptoethanol (TR buffer), and the enzyme (50 μg protein). Production of elemental tellurium was monitored at 500 nm using a Tecan Infinite^®^ M200 PRO plate reader. One enzyme unit was defined as the amount of enzyme required to increase the OD_500_ by 0.001 in 1 min under the assay conditions as described earlier (Chiong et al., 1988; Castro et al., 2008; Arenas et al., 2014a). The effect of pH on tellurite reduction was assessed by determining TR activity at 37°C using the following buffers at 50 mM: Na_2_HPO_4_-citric acid (pH 3.0–6.0), Tris-HCl (pH 7.0–9.0), glycine/NaOH (pH 10.0), carbonate/NaOH (pH 11.0) and KCl/NaOH (pH 12.0). The effect of temperature on TR activity was determined at the optimal pH for each enzyme in a temperature range that included 25, 30, 37, 42, and 50°C. The apparent kinetic parameters were determined in triplícate using the same reaction mixture as for tellurite reduction at pH and temperature optima for each enzyme; tellurite concentrations varied from 0 to 2 mM. Maximal velocity, apparent *K*_m_ and *K*_i_ were determined by fitting non-linear regression using the GraphPad Prism Version 7.01 program (**Table 1**).

### Synthesis and Characterization of TeNS

*In vivo* synthesis of TeNS was performed using *E. coli* (ASKA collection, Supplementary Table S1) grown to exponential phase (OD_600_ ∼ 0.3), induced with 1 mM IPTG, treated with 0.5 μg/ml TeO_3_^2-^ for 4 h and centrifuged at 6,000 × *g* for 10 min. The bacterial pellet was sent to the Advanced Microscopy Unit (AMU) at Pontificia Universidad Católica de Chile for thin sectioning and transmission electron microscopy (TEM) analysis. *In vitro* TeNS production was carried out for 60 min in TR buffer at pH and temperature optima using 250 μg/ml of purified enzyme. Samples were analyzed by TEM using a Philips Tecnai 12 TEM.

The hydrodynamic diameter of TeNS (*in vitro* synthesis) was determined at room temperature (25°C) using a Zetasizer Nano ZS Marlvern instrument. Values were calculated from three independent measurements of 20 repetitions each. Tellurium in TeNS generated *in vitro* was quantified by optical emission spectrometry-inductively coupled plasma (ICP-OES) using a Perkin Elmer 2000 DV optimum with a wavelength of 214.281 nm corresponding to tellurium, as described previously (Pugin et al., 2014). A calibration curve (1–200 μg/ml) in 2% ultrapure HNO_3_ was constructed using pure, commercially available tellurium (Sigma–Aldrich). *In vitro*-generated samples were sedimented (13,000 × *g* for 90 min), washed two times with milliQ water and suspended in 10% HNO_3_. Once completely solubilized, they were filtered through 0.2 μm pore membranes and analyzed by ICP-OES.

## Results

### Identifying Putative Tellurite Reductases

As mentioned, several enzymes able to reduce tellurite have been identified. In this line, it is intriguing that enzymes catalyzing very different biological reactions are capable of tellurite reduction. These proteins show very low amino acid sequence identity (<30%) and no obvious conserved motifs.

To look for domains and/or functional sites that may be common to these proteins, they were independently characterized using bioinformatic resources that included Prosite (Sigrist et al., 2013), InterPro (Mitchell et al., 2014), SCOP (Andreeva et al., 2008), CATH (Sillitoe et al., 2013), and Pfam (Finn et al., 2014). InterPro and Pfam showed the presence of two groups of proteins, one exhibiting the pyridine nucleotide-disulphide oxidoreductase [FAD/NAD(P)-binding] domain (characteristic of flavoproteins) and the second exhibiting the molybdopterin oxidoreductase 4Fe–4S domain, which is found in a number of reductase/dehydrogenase families (Supplementary Table S2).

The Prosite database allowed identification of the pyridine nucleotide-disulphide oxidoreductase class-I active site (PS00076), encompassing the amino acid pattern G-G-x-C-[LIVA]-x(2)-G-C-[LIVM]-P, and also the class-II active site C-x(2)-C-D-[GAS]-x(2,4)-[FYA]-x(4)-[LIVMAT]-x(0,1)-[LIVM](2)-[GI]-[GDS]-[GRD]-[DN] (PS00573). Another interesting organization found in some of these proteins was the “ferredoxin-type iron-sulfur binding” domain, which displays the pattern C-x-{P}-C-x(2)-C-{CP}-x(2)-C-[PEG]. All the above mentioned domains contain nearby cysteine residues at the active site, which play a critical role in catalysis (see below).

Since it was previously shown that flavoproteins such as GorA (Pugin et al., 2014) and E3 (Castro et al., 2008) display TR activity, we decided to carry out a more in-depth analysis of the FAD/NAD(P)-binding and pyridine nucleotide-disulphide oxidoreductase domains as being -at least- partly responsible for tellurite reduction. Using these domains as a signature pattern to identify putative TR enzymes, a cross-search for them in *E. coli* proteins was carried out. Utilizing different data bases (Uniprot, SCOP, PFAM, Prosite) a number of enzymes bearing the referred motifs were identified. Eight of them, namely TrxB, AhpF, glutamate synthetase (GltD), putative oxidoreductase (YkgC), GorA, nitrite reductase (NirB), flavorubredoxine reductase (NorW), and mercury reductase (MerA) were selected for further analysis. Unfortunately, we were unable to purify NirB and GltD thus hampering their characterization.

Given that numerous enzymes display these motifs, starting point was defined strains from the *E. coli* ASKA collection that overproduced enzymes exhibiting vicinal Cys residues as well as the FAD/NAD(P) motif; these were then used to purify enzymes to be tested for TR activity (see below). Enzymes lacking the FAD and/or NAD(P)^+^ binding domains or the catalytic cysteines (SthA, PreT, PreA, HcaD, and Glf) were also tested for TR activity. Unfortunately, we were unable to purify NirB and GltD thus hampering their characterization.

### Purification of Flavoproteins Predicted to Display TR Activity

Six of the above proteins (TrxB, AhpF, YkgC, GorA, E3, and NorW) were purified after being overproduced in *E. coli* (Supplementary Table S1, **Supplementary Figure S1A**). Since *E. coli* lacks *merA*, it was amplified from the environmental plasmid pTP6 (Smalla et al., 2006), cloned and over- expressed in this bacterium (Supplementary Table S1, **Supplementary Figure S1B**). To assess if there were differences between TR enzymes from tellurite-sensitive (*E. coli*) and tellurite-resistant (*S. haemolyticus* BNF01; Arenas et al., 2014b) organisms, the *ahpF* and *trxB* genes from this strain were cloned, over-expressed and the respective proteins purified (Supplementary Table S1, **Supplementary Figure S1C**). Excepting for NorW (**Supplementary Figure S1A**, lane 7), all other proteins were obtained with a purity of >90%, as judged from denaturing polyacrylamide gel electrophoresis.

### TR Activity of *E. coli* Flavoproteins

Purified proteins were assessed for TR activity in the presence of NADH or NADPH as electro donor. While GorA, E3, and TrxB used preferentially NADPH, AhpF, YkgC, and NorW used NADH as cofactor (**Table 1**). Next, the effect of pH and temperature on TR activity was determined for each flavoprotein using the preferred pyridine cofactor.

**FIGURE 1:**
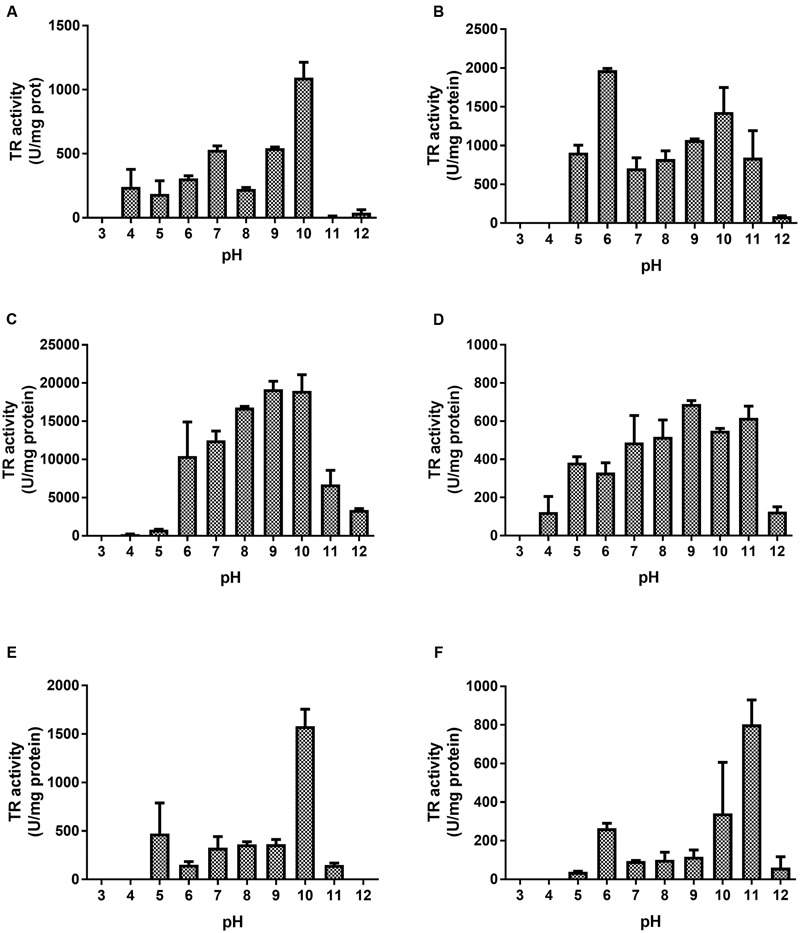
**Effect of pH on the TR activity of *E. coli* flavoproteins.** The effect of pH on tellurite reduction was assessed by determining TR activity at different pH values [Na_2_HPO_4_-citric acid (pH 3.0–6.0), Tris-HCl (pH 7.0–9.0), glycine/NaOH (pH 10.0), carbonate/NaOH (pH 11.0), and KCl/NaOH (pH 12.0)]. **(A)** AhpF, **(B)** E3, **(C)** GorA, **(D)** YkgC, **(E)** TrxB, **(F)** NorW. Data represent the average of three independent trials ±SD.

**FIGURE 2:**
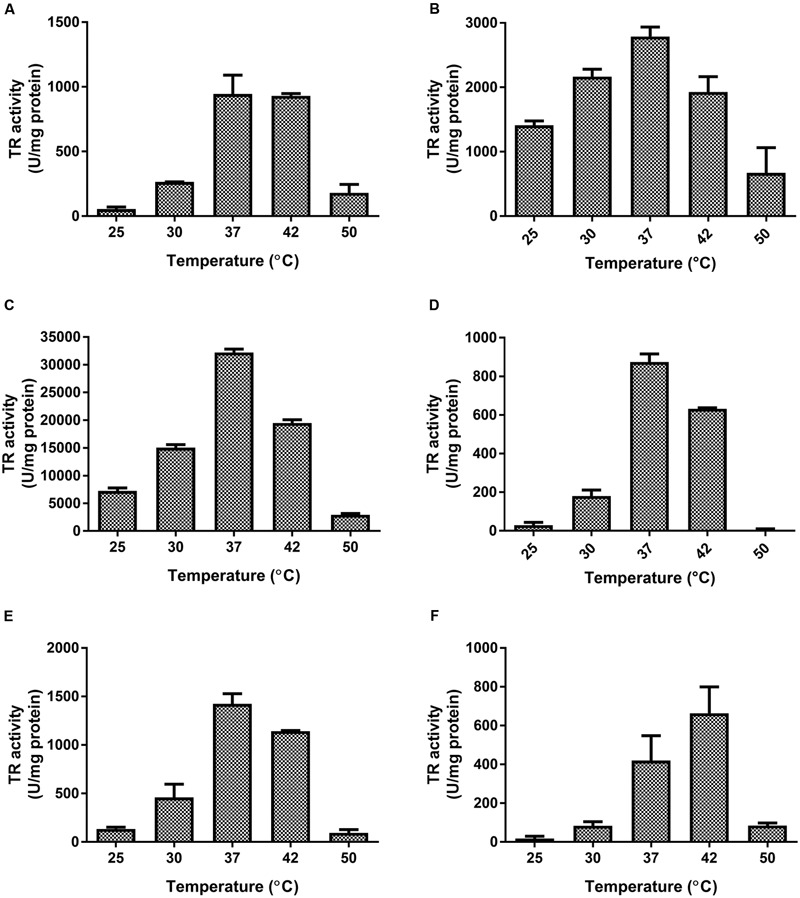
**Temperature dependence of TR activity from *E. coli* flavoproteins.** TR activity was determined at the optimal pH for each enzyme. **(A)** AhpF, **(B)** E3, **(C)** GorA, **(D)** YkgC, **(E)** TrxB, **(F)** NorW. Data represent the average of three independent trials ±SD.

Most enzymes showed maximal tellurite reduction at pH 8.0–10.0 (**Figure 1**); the exception was E3, which showed maximal TR activity at a rather acidic pH (**Figure 1B**). While GorA exhibited the highest TR activity (∼30,000 U/mg protein, **Figure 2C**), NorW and YkgC displayed the lowest (∼660 and 870 U/mg protein, respectively; **Table 1**). Tellurite-reducing activity was extremely low and almost undetectable at pH 3.0–4.0 (**Figure 1**). As expected, PreT, PreA, HcaD, and Glf did not show TR activity under these conditions (not shown).

The effect of temperature was assessed for GorA, AhpF, YkgC, TrxB, NorW, and E3. At their optimal pH values, all of them exhibited a similar behavior in the range of 25–50°C, with peak activity at ∼37°C (**Figure 2**). GorA showed ∼11.5-, 22.6-, 34.5-, 36.8-, and 48.5-fold more tellurite-reducing activity than E3, TrxB, AhpF, YkgC, and NorW, respectively (**Table 1**). Kinetic parameters such as *K*_m_, *K*_i_, and *V*_max_ are shown in **Table 1**. While AhpF, YkgC, and NorW showed Michaelis–Menten kinetics, GorA, E3, and TrxB exhibited a behavior compatible with substrate (tellurite) inhibition (not shown). As expected, enzymes exhibiting higher TR activity displayed lower *K*_m_ and higher *V*_max_ values.

### TR Activity of Flavoproteins from the Tellurite-Resistant, Environmental *Staphylococcus* BNF01 Strain, and the pTP6 Plasmid-Encoded MerA

**FIGURE 3:**
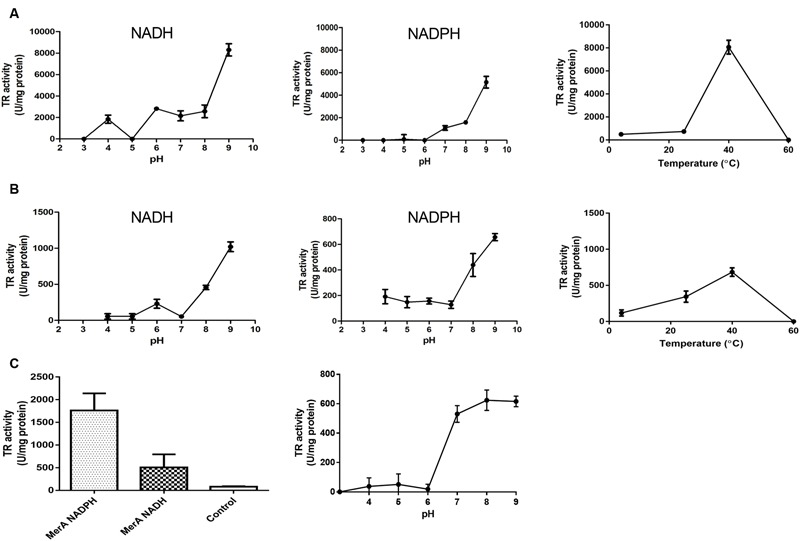
**TR activity of AhpF and TrxB from *Pseudomonas* spp. BNF01 and MerA from pTP6 plasmid.** The activity of AhpF **(A)** and TrxB **(B)** was assayed in the presence of NADH or NADPH at the indicated pH and temperature values. **(C)** Specific TR activity of MerA in the presence of the indicated cofactors at pH 7.0 (left); effect of pH on tellurite reduction (right); controls contained no enzyme. Data represent the average of three independent trials ±SD.

*Staphylococcus haemolyticus* BNF01 genes encoding TrxB and AhpF were cloned and the proteins purified to determine if they displayed TR activity. TrxB and AhpF displayed maximal NADH-dependent TR activity at pH 9.0 and 40°C (**Figures 3A,B**). As with GorA, AhpF, YkgC, TrxB, NorW, and E3, tellurite reduction by TrxB and AhpF from BNF01was inhibited by divalent metals such as Zn^2+^, Ni^2+^, and Co^2+^ (not shown). In turn, pTP6-encoded MerA showed TR activity at pH 7.0–9.0 in the presence of NADPH (**Figure 3C**).

### *In Silico* Analysis of *E. coli* Flavoproteins Exhibiting TR Activity

The primary amino acid sequence of these flavoproteins was examined using the PROSITE database. Three common pattern groups were identified: PS00573, PS51354, and PS00076. TrxB and AhpF displayed a similar active site, with the class-II pyridine nucleotide-disulphide oxidoreductase, and GorA, E3, and YkgC with the class-I pyridine nucleotide-disulphide oxidoreductases (Supplementary Table S2).

**FIGURE 4:**
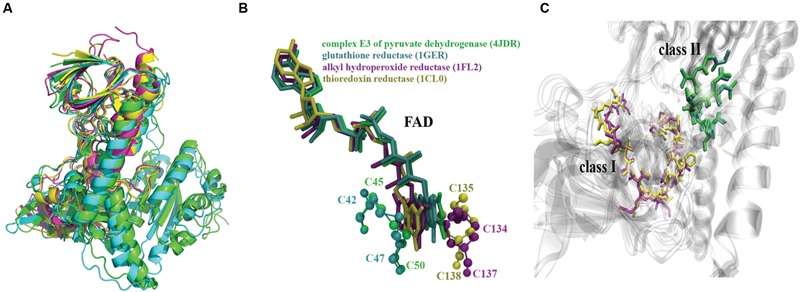
***In silico* analysis of *E. coli* TR flavoproteins.** E3 (PDB_ID: 4JDR, green), GorA (PDB_ID: 1GER, cyan), AhpF (PDB_ID: 1FL2, purple), and TrxB (PDB_ID: 1CL0, yellow) flavoproteins are shown. **(A)** Structure alignment of these proteins showing their 3D structure available in PDB data base (Raptor X software; Wang S. et al., 2011). The global RMSD was 3.61 Å. **(B)** Structure alignment for the FAD molecule and representation of the interaction with the two catalytic cysteine residues of each protein. **(C)** Representation of the pyridine nucleotide-disulphide oxidoreductases class-I and class-II active sites.

In spite that the amino acid sequence of the two classes of enzymes showed low identity (**Supplementary Figure S2**), they actually did exhibit high structural similarity exhibiting a global RMSD of 3.61 Å (**Figure 4A**). Although the SSAP score within proteins of the same class was close to 90 points, that of proteins of different classes is still high (72 points; Supplementary Table S3).

In all cases, the FAD molecule was bound to the active site by the vicinal cysteine residues and adopted a similar conformation (**Figures 4B,C**). Although both motifs I and II were characterized using FAD to convey reducing power, the spatial localization of the disulfide bridge was different between them (**Figures 4B,C**, Supplementary Table S4).

**FIGURE 5:**
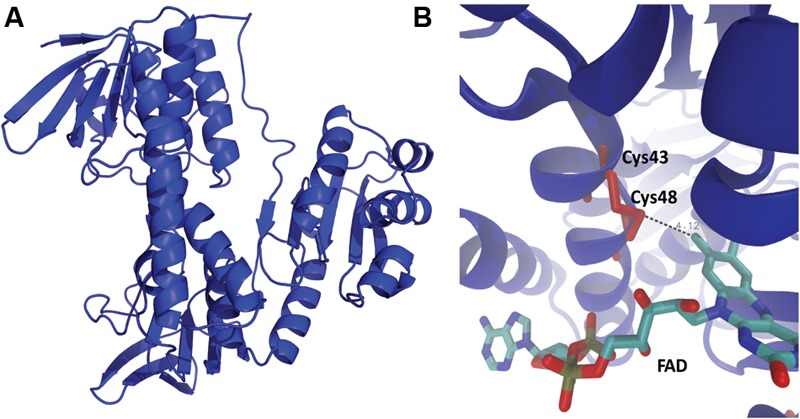
**Structural model of *E. coli* YkgC. (A)** 3D structure of the protein was built through comparative modeling as described in Section “Materials and Methods.” **(B)** Protein-FAD complex was predicted by docking simulations. YkgC is shown in blue while Cys43 and Cys48 are depicted in red. The FAD molecule is shown by licorice representation.

It is intriguing that to date no biological function has been reported for *E. coli* YkgC, one of the flavoenzymes exhibiting TR activity. Since there are no data regarding its spatial structure, the 3D structure of the protein was built by means of comparative modeling, which resulted in a configuration very similar to that of dihydrolipoyl dehydrogenase (4JDR), with a SSAP score of 89.6 (Supplementary Table S3). Although not exactly in the same position, FAD was predicted to bind to the same zone as in the other flavoproteins, with the flavin ring located near to the two cysteines (**Figure 5**), as predicted by docking (Autodock software).

Next, the relevant distances at the active site of the flavoproteins were determined from the 3D structure. Supplementary Table S4 summarizes the distances that are predicted to be important for the displayed TR activity. In general, shorter distances between cysteine residues were associated with higher TR activity (distance 4, Supplementary Table S4), as it occurs in GorA and E3. This could be even more relevant for TR activity that interaction between cysteines and the FAD moiety (distances 2, 3, 5, Supplementary Table S4).

### Correlations of TR Activity-TeNS Synthesis by Flavoproteins

The synthesis of tellurium-based nanostructures is a relatively new field that has gained interest because of the multiple potential applications of the TeNS. Along this line, after characterizing TR activity, it was very interesting to analyze the reduction products. *In vitro* synthesis of nanostructures was carried out using GorA, YkgC, E3, and AhpF, as described in Section “Materials and Methods.” Dynamic light scattering was used to determine the hydrodynamic diameter of the *in vitro* generated TeNS.

**FIGURE 6:**
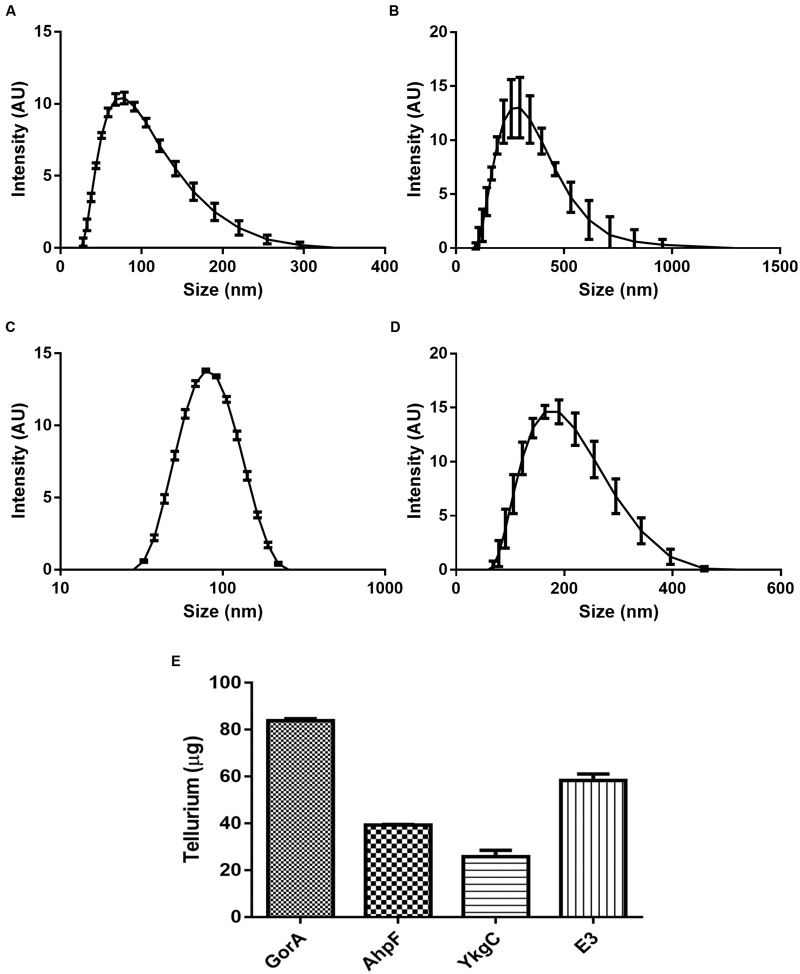
**Characterization of *in vitro* synthesized TeNS.** Nanostructures synthesized using *E. coli* flavoproteins AhpF **(A)**, E3 **(B)**, GorA **(C)** or YkgC **(D)** were subjected to dynamic light scattering analysis. The amount of tellurium present in nanostructures was determined by ICP-OES **(E)**. Data represent the average of three independent trials ±SD.

Structures generated by E3 showed a wide size distribution, from a few nanometers to micrometers. Although with large deviations, the most abundant structure observed had a hydrodynamic diameter of 254.2 nm, with an average PDI (polydispersity index) of 0.159 ± 0.041 (**Figure 6B**).

Structures synthesized using GorA exhibited a Gaussian size distribution (**Figure 6C**). The most abundant nanostructure showed a maximum hydrodynamic diameter of 70.37 nm (PDI 0.217 ± 0.004). Similarly, AhpF (**Figure 6A**) generated structures of 78.06 nm (PDI 0.345 ± 0.013). However, their size distribution showed a smoother attenuation at larger sizes, resulting in a higher number of structures exceeding 200 nm. On the other hand, tellurium structures generated by YkgC showed a size distribution that was similar to that generated by E3, with a peak at 162.2 nm and a PDI of 0.125 ± 0.011 (**Figure 6D**).

To determine if there is a correlation between TR activity and the amount of produced NS, the amount of tellurium present in the NS was quantified by ICP-OES. In general, Te content of NS correlated well with TR activity (**Table 1**, **Figure 6E**). For instance, TeNS produced with GorA contained 3.25 times as much Te as those produced with YkgC (**Figure 6E**).

**FIGURE 7:**
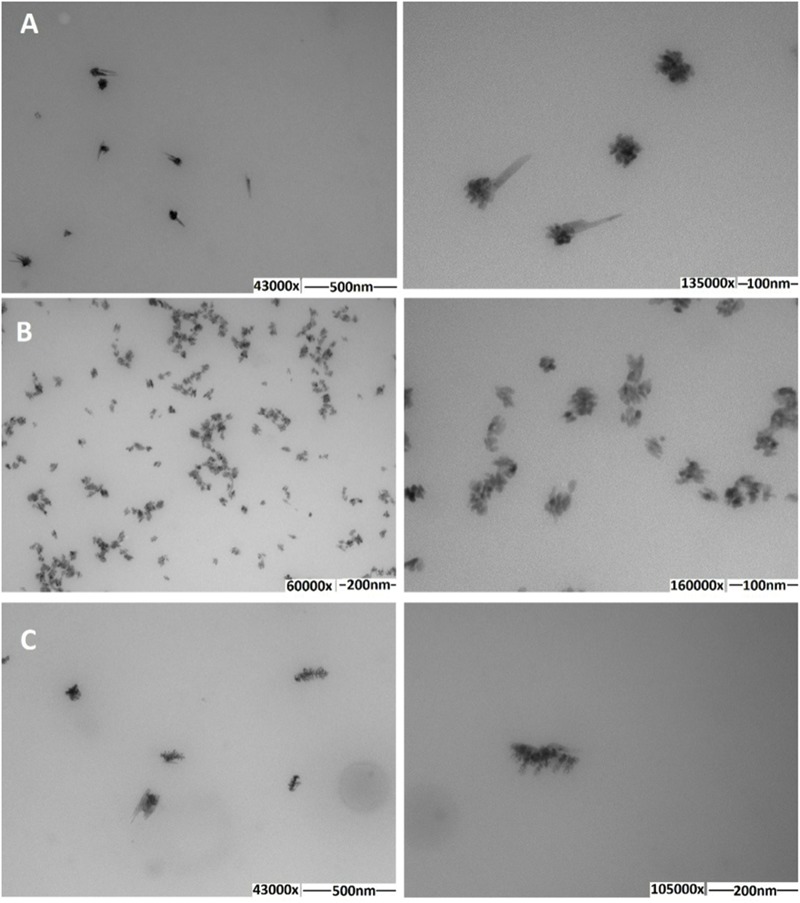
**Transmission electron microscopy of flavoprotein-synthesized TeNS.** Structures resulting from the *in vitro* tellurite reduction by *E. coli* flavoproteins AhpF **(A)**, GorA **(B)**, and YkgC **(C)** were visualized by TEM as described in Section “Materials and Methods.”

Te-containing nanostructures synthesized using GorA, YkgC, and AhpF were analyzed by TEM (**Figure 7**). The results showed that they varied in size and morphology depending on the synthesizing enzyme. In this line, AhpF generated rather rounded structures of 50–100 nm with irregular edges that doubled the size of the center core (**Figure 7A**, right). On the other hand, GorA-synthesized TeNS exhibited a compact morphology with a roundish shape and amorphous edges. Size and shape differences did occur, maintaining the general characteristics described above (**Figure 7B**). Structures produced by YkgC showed larger average sizes, often exceeding 100 nm. The morphology was rather elongated and irregular rods with numerous tips outlining its contour were seen (**Figure 7C**).

To determine if such particles were also synthesized *in vivo*, the synthesis of TeNS was assessed by electron microscopy in ultrathin sections of *E. coli* overproducing tellurite reductases (TRs; **Supplementary Figure S3**). Membrane damage and electron-dense elements (probably Te^0^-containing structures) were seen only in tellurite-exposed cells that over synthesized GorA, YkgC, or TrxB (**Supplementary Figures S3C–E**). As seen *in vitro*, strains overproducing YkgC also exhibited larger electron-dense structures (**Supplementary Figure S3D**).

Finally, electron-dense elements were not observed in cells overproducing AhpF and E3, indicating that TR activity does not necessarily correlate with the generation of electron-dense deposits *in vivo* (**Supplementary Figures S3A,B**).

## Discussion

Predicted proteins exhibiting tellurite reducing activity -in spite of their low amino acid sequence identity (∼20%)- did share the G-X(1-2)-G-X-X-G NAD(P)H-binding motif. Analysis of secondary structure indicated that most of them adopt a common structural Rossmann folding domain βαβαβ (Rossmann et al., 1974), a nucleotide-binding motif that is characteristic of oxidoreductases. These proteins possess catalytic redox sites that accommodate the substrate NAD(P)H, which is involved in tellurite reduction (Castro et al., 2008; Arenas et al., 2014a). Multiple sequence alignments showed some conserved amino acids such as tyrosine, aspartic acid, glutamic acid, and cysteine, which could participate in electron transfer (Brömme et al., 2002) and hence, in tellurite reduction.

Using different databases such as Pfam, CATH, and SCOP to analyze TR proteins, we found some common domains like the FAD/NAD(P)^+^-binding motif that belongs to a particular group of flavoprotein disulfide reductases (FDR). In general, FDR represents a family of enzymes that share high sequence and structural similarity (Argyrou and Blanchard, 2004). With the aim to find new TR enzymes exhibiting (i) the common structural domains in TR proteins (PF02852, PF0070, and PF07992), (ii) the presence of a FMN or FAD binding site, and (iii) the presence of vicinal cysteine residues were used to subdivide the *E. coli* flavoprotein family. As a result, various potential tellurite-reducing enzymes (TrxB, AhpF, YkgC, GorA, NirB, E3, GltD, and NorW) were identified. Only E3 from *Aeromonas caviae* (Castro et al., 2008; Arenas et al., 2014a), TrxR and Gor from rat liver (Rigobello et al., 2004), and Gor from the antarctic *P. lini* BNF22 strain (Pugin et al., 2014) were previously reported to display the ability to reduce tellurite.

In this study, most of the purified proteins exhibited tellurite-reducing activity at the same temperature (**Figure 2** and **Table 1**), thus validating the bioinformatic approach used for their identification.

Regarding the electron donor, only dihydrolipoamide dehydrogenase exhibited preference for NADPH instead of its normal cofactor NADH (**Table 1** and Supplementary Table S5), a situation that could occur because of the known inhibition of E3 by NADH (Schmincke-Ott and Bisswanger, 1981). Furthermore, and as expected, enzymes lacking the FAD/NAD(P)^+^-binding domain or the catalytic cysteines (SthA, PreT, PreA, HcaD, and Glf), did not show tellurite-reducing activity. These were first considered because they exhibited some of the motifs forming part of the active site of TRs. Further 3D as well as molecular simulation analysis could help to explain the lack of TR activity in these proteins.

The large amino acid sequence homology of enzymes belonging to the FDR family (Argyrou and Blanchard, 2004) could in part explain the similar optimal pH for tellurite reduction (**Figure 1**). Crystallographic data showed high similarity between GorA and E3 active sites. Both proteins share motifs such as the active disulfide C-N-X-X-X-X-C-C and the pair interface H-X-X-X-X-E, which holds FAD and NAD(P)H binding domains. As previously shown, the catalytic activity of Cys residues is crucial for E3’s TR activity (Arenas et al., 2014a). These residues are pH-sensitive, and as opposed to the highly reactive thiolate anion (S^-^), thiol groups are not good nucleophiles (Vlamis-Gardikas, 2008). These traits could underlie the higher activity exhibited by most TRs at rather basic pH values.

An aspartic acid residue near the active site of TrxB would act as an acid–base catalyst that at basic pH would favor cysteine deprotonation thus increasing its catalytic activity (**Figure 1E**) (Mulrooney and Williams, 1994). The activity exhibited by E3 and NorW at pH 6.0 (**Figures 1B,F**) could be explained by the existence of two redox CXXXXC centers in which cysteine residues display different pKa values, one of them being 6.3 (Wood et al., 2001). At this pH, a hydrogen bond would form between cysteine residues which in turn would stabilize thiolate formation at the other redox center, which then could function in catalysis.

Recombinant AhpF and TrxB from *S. haemolyticus* BNF01 showed the highest TR activity at pH 9.0 and 40°C using NADH as electron donor (**Figures 3A,B**). Furthermore, both enzymes were inhibited by divalent cations such as Zn^2+^, Ni^2+^, and Co^2+^, suggesting the importance of cysteine in TR activity. The *E. coli* orthologs exhibited the same optimal pH and temperature, although *E. coli* TrxB uses NADPH (Seaver et al., 2001; Lu and Holmgren, 2014) instead of NADH as electron donor (**Figure 3B**). The choice of the enzyme for NADH or NADPH would apparently be the result of cofactor stabilization at the protein active site (Bellamacina, 1996; Rigobello et al., 2004).

Of the enzymes displaying TR activity, those containing FAD are the most effective in reducing the tellurium oxyanion. In this context, an *in silico* analysis indicated that MerA should be a potential TR. Since *E. coli* lacks the *merA* gene, we were prompted to clone it from the environmental plasmid pTP6, which harbors the *merRTPGABDE* operon (Smalla et al., 2006). As expected, purified MerA efficiently reduced tellurite at pH 7.0–9.0 at 37°C using NADPH as electron donor (**Figure 3C**).

It was conceived that higher TR activities should result from shorter distances between atoms participating in catalysis at the active site (Supplementary Table S4). For instance, AhpF displays the shortest distance between cysteine thiols and FAD’s carbon 4, which could facilitate electron transfer among them. Likewise, GorA displays the shortest distance between the Cα of cysteine residues while E3 shows the shortest distance between the thiol groups that form the disulfide bridge (Supplementary Table S4); these shorter distances might facilitate disulfide bond breaking, thus favoring TR activity.

On the other hand, using enzymes to synthesize metal(loid)-containing NS is a relatively new process. Along this line, only the enzymatic synthesis of silver- (Kumar et al., 2007a), gold- (Kumar et al., 2007b; Scott et al., 2008), cadmium- (Ansary et al., 2007), tellurium- (Monrás et al., 2014), nickel-, lead-, and cobalt-containing nanostructures (Ansary et al., 2012) has been described. So far, TeNS chemical synthesis to form nanopencils, nanorices, nanowires, and nanocubes has been reported (Lin et al., 2012). These are of biotechnological interest because of their antibacterial properties, which are equal or greater than that of silver nanoparticles. Moreover, because of their improved reactivity they have been used to manufacture nitric oxide (Kumar et al., 2009) and chlorine (Sen et al., 2009) sensors. Unfortunately, protocols for chemical synthesis of TeNS involve high temperatures, toxic reagents and anaerobic conditions, thus affecting the clinical applications of the nanostructures. The search for environmentally friendly and economical methods has made biological systems attractive candidates for synthesizing nanostructures *in vivo* and *in vitro*. In this context, the bacterial synthesis of (i) tellurium nanospheres by *Sulfurospirillum barnesii* (Baesman et al., 2007) and *P. pseudoalcaligenes* KF707 (Di Tomaso et al., 2002), (ii) tellurium nanorods by *Bacillus selenitireducens* (Baesman et al., 2007) and *E. coli* (Wang X. et al., 2011), (iii) CdTe quantum dots by *E. coli* (Bao et al., 2010; Park et al., 2010) and some yeasts (Ba et al., 2010) has been documented. More recently, the *in vitro* synthesis of Te-containing nanostructures using GorA from the Antarctic strain *Pseudomonas* sp. BNF22 has been reported (Pugin et al., 2014).

Since *E. coli* GorA, E3, AhpF, and YkgC displayed TR activity (**Table 1**), they were used to synthesize tellurium-containing assemblies *in vitro*. Structures generated by GorA and AhpF (**Figures 6** and **7**) were within the expected size range of nanostructures, i.e., not exceeding 100 nm. Conversely, structures produced by YkgC and E3 were above 100 nm and thus cannot be considered NS. Although the enzymatic synthesis of TeNS reported here did not generate well defined structures, it seems that each protein could function in synthesizing TeNS that exhibit a defined morphology.

Finally, *E. coli* strains over-expressing tellurite-reducing enzymes generated electron-dense intracellular deposits that were less abundant and smaller in size than those generated *in vitro* by purified TR enzymes (**Supplementary Figure S3**). Although these enzymes exhibit high TR activity *in vitro*, their activity cannot be compared to that *in vivo*, because multiple factors influence the activity in the cell and, thus, in NS production. Further studies to determine both the mechanism(s) of NS synthesis and the factors influencing NS shape, growth, and structure are being pursued in our laboratory.

## Conclusion

Characteristic structural domains of tellurite-reducing enzymes were identified by *in silico* analysis. These allowed the identification of new flavoproteins displaying TR activity. The key components for reduction are two catalytic cysteine residues and NAD(P)H- and FAD-binding motifs as electron donors. Defined flavoproteins exhibiting TR activity generated tellurium-based nanostructures *in vitro*.

## Author Contributions

Conceived and designed the experiments: MA-S, JV-P, EM, CV, and FA. Performed the experiments: MA-S, JV-P, WM, CP, PM-D, FC, BP, CM-V, and FR-R. Analyzed the data: MA-S, JS, WD-V, JS, CV, and FA. Contributed reagents/materials/analysis tools: MA-S, CV, and FA. Wrote the paper: CV and FA.

## Conflict of Interest Statement

The authors declare that the research was conducted in the absence of any commercial or financial relationships that could be construed as a potential conflict of interest.

## Footnotes

1 http://www.ncbi.nlm.nih.gov/nuccore/AM048832.1

## Supplementary Material

FIGURE S1 **SDS-PAGE of purified putative tellurite reductases. (A)** Purified *E. coli* flavoproteins E3 (∼56 kDa), YkgC (∼48 kDa), GorA (∼49 kDa), AhpF (∼56 kDa), TrxB (∼35 kDa), SthA (∼52 kDa), and NorW (∼41 kDa) were visualized by PAGE-SDS. Each lane contained 15 μg of protein. **(B)** Mercuric reductase (MerA, ∼56 kDa, 10 μg) from the environmental plasmid pTP6. **(C)** *S. haemolyticus* BNF01 flavoproteins AhpF (∼60 kDa, 10 μg) and TrxB (∼35 kDa, 20 μg). Protein standards (St) were SDS7B2 Sigma **(A)**, Broad Range NEB **(B)** and MW High-range Gibco BRL.

FIGURE S2 **Amino acid sequence alignment of the indicated *E. coli* flavoproteins.** Secondary structure elements of E3 (PDB_ID: 4JDR), GorA (PDB_ID: 1GER), AhpF (PDB_ID: 1FL2), and TrxB (PDB_ID: 1CL0) are indicated at the top (hairsprings: α-helix; arrows, β-sheets). Cys residues are indicated by black dots.

FIGURE S3 **Transmission electron microscopy of *in vivo* synthesized electron-dense assemblies.** The structures resulting from the *in vivo* tellurite reduction by *E. coli* strains overproducing AhpF **(A)**, E3 **(B)**, GorA **(C)**, YkgC **(D)**, TrxB **(E)**, and NorW **(F)** were visualized by TEM as described in Section “Materials and Methods.”
