# Supplementary material for: Flavoprotein-Mediated Tellurite Reduction: Structural Basis and Applications to the Synthesis of Tellurium-Containing Nanostructures
Source: Front Microbiol. 2016 Jul 26;7:1160. doi: 10.3389/fmicb.2016.01160 (PMC4960239; doi:10.3389/fmicb.2016.01160)
Supplement: Supplementary file 1 [file Table_1.DOCX]

Supplementary Material

**Flavoprotein-mediated tellurite reduction: structural basis and applications to the synthesis of tellurium-containing nanostructures**

Mauricio Arenas-Salinas, Joaquín Vargas-Pérez, Wladimir Morales, Camilo Pinto, Pablo Muñoz, Fabián Cornejo, Benoit Pugin, Juan Sandoval, Waldo Díaz-Vásquez, Claudia Muñoz-Villagrán, Fernanda Rodríguez-Rojas, Eduardo Morales, Claudio C. Vásquez, Felipe Arenas

**Correspondence to:** Felipe A. Arenas and/or Claudio C. Vásquez. E-mails: [felipe.arenass@usach.cl](mailto:felipe.arenass@usach.cl); [claudio.vasquez@usach.cl](mailto:claudio.vasquez@usach.cl)

**Table 1S. Bacterial strains and plasmids**

| *E. coli* strain | Relevant genotype | Source or reference |
| --- | --- | --- |
| AG1 | Wild-type, *recA*1 *endA*1 *gyrA*96 *thi*-1 *hsd*R17(rK-mK+) *sup*E44 *rel*A1 | NARA Institute, Japan |
| ASKA *gorA* | AG1 harboring pCA24N/*gorA.* Cam^R^ | NARA Institute, Japan |
| ASKA *ahpF* | AG1 harboring pCA24N/*ahpF.* Cam^R^ | NARA Institute, Japan |
| ASKA *trxB* | AG1 harboring pCA24N/*trxB.* Cam^R^ | NARA Institute, Japan |
| ASKA *ykgC* | AG1 harboring pCA24N/*ykgC.* Cam^R^ | NARA Institute, Japan |
| ASKA *norW* | AG1 harboring pCA24N/*norW.* Cam^R^ | NARA Institute, Japan |
| ASKA *lpdA* | AG1 harboring pCA24N/*lpdA.* Cam^R^ | NARA Institute, Japan |
| ASKA *sthA* | AG1 harboring pCA24N/*sthA.* Cam^R^ | NARA Institute, Japan |
| ASKA nirB | AG1 harboring pCA24N/*nirB.* Cam^R^ | NARA Institute, Japan |
| BL21 | F^-^*omp*T *hds*S_B_ (r_B_^-^m_B_^-^) *gal dcm rne*131 (DE3) | James Imlay |
| BL21 pET/*trx*B2 | BL21 harboring pET/*trx*B | This work |
| BL21 pET/*ahp*F2 | BL21 harboringpET/*ahp*F | This work |
| BL21 pET/*merA* | BL21 harboring pET/*merA* | This work |
| Plasmids | **Relevant characteristics** | **Source or reference** |
| pET 101/D TOPO | Expression vector, Amp^R^ | INVITROGEN^®^ |
| pET/*trxB* | pET101 harboring the BNF01 *trxB* gene*.* Amp^R^ | This work |
| pET/*ahpF* | pET101 harboring the BNF01 *ahpF* gene. Amp^R^ | This work |
| pET/ *merA* | pET101 harboring the pTP6 *merA* gene. Amp^R^ | This work |
| pCA24N | Expression vector, Cam^R^ | Kitagawa *et al*., 2005 |
| pCA24N/*gorA* | pCA24N harboring the *E. coli gorA* gene*.* Cam^R^ | NARA Institute, Japan |
| pCA24N/*ahpF* | pCA24N harboring the *E. coli ahpF* gene*.* Cam^R^ | NARA Institute, Japan |
| pCA24N/*trxB* | pCA24N harboring the *E. coli trxB* gene*.* Cam^R^ | NARA Institute, Japan |
| pCA24N/*ykgC* | pCA24N harboring the *E. coli ykgC* gene*.* Cam^R^ | NARA Institute, Japan |
| pCA24N/*norW* | pCA24N harboring the *E. coli norW* gene*.* Cam^R^ | NARA Institute, Japan |
| pCA24N/*lpdA* | pCA24N harboring the *E. coli lpdA* gene*.* Cam^R^ | NARA Institute, Japan |
| pCA24N/*sthA* | pCA24N harboring the *E. coli sthA* gene*.* Cam^R^ | NARA Institute, Japan |
| pCA24N/*nirB* | pCA24N harboring the *E. coli nirB* gene*.* Cam^R^ | NARA Institute, Japan |
| Primers | **Forward (F) and reverse (R) primers to** | **5’- 3’ Sequence ^** |
| *ahp*F F | BNF01 *ahpF* gene*,* forward | CACCATGCTTAATGCTGATTTAAAACAAC |
| *ahp*F R | BNF01 *ahpF* gene*,* reverse | GTTTCTAATGATATAATCAAATGCA |
| *trx*B F | BNF01 *trxB* gene*,* forward | CACCATGACTGAAGTAAATTATGATGTTGC |
| *trx*B R | BNF01 *trxB* gene*,* reverse | TGCTTCTTGCTTATCTTTTAATTCTTC |
| *merA* F | pTP6 *merA* gene, forward | CACCATGCAAGAGTTAAACGAAGTTGGT |
| *merA* R | pTP6 *merA* gene, reverse | GCCGGCACAGCACGATAGCT |
| pET F | PET TOPO 101, forward | ATGCGTCCGGCGTAGAGG |
| pET R | PET TOPO 101, reverse | GCTAGTTATTGCTCAGCGGTGG |
