# Supplementary material for: Flavoprotein-Mediated Tellurite Reduction: Structural Basis and Applications to the Synthesis of Tellurium-Containing Nanostructures
Source: Front Microbiol. 2016 Jul 26;7:1160. doi: 10.3389/fmicb.2016.01160 (PMC4960239; doi:10.3389/fmicb.2016.01160)
Supplement: Supplementary file 2 [file Table_2.DOC]

Supplementary Material

**Flavoprotein-mediated tellurite reduction: structural basis and applications to the synthesis of tellurium-containing nanostructures**

Mauricio Arenas-Salinas, Joaquín Vargas-Pérez, Wladimir Morales, Camilo Pinto, Pablo Muñoz, Fabián Cornejo, Benoit Pugin, Juan Sandoval, Waldo Díaz-Vásquez, Claudia Muñoz-Villagrán, Fernanda Rodríguez-Rojas, Eduardo Morales, Claudio C. Vásquez, Felipe Arenas

**Correspondence to:** Felipe A. Arenas and/or Claudio C. Vásquez. E-mails: [felipe.arenass@usach.cl](mailto:felipe.arenass@usach.cl); [claudio.vasquez@usach.cl](mailto:claudio.vasquez@usach.cl)

Table 2S. Functional domains present in enzymes displaying TR activity.

| **Protein** | **Gene** | **Organism** | **InterProScan - Domain** | **Pfam** | **Prosite** |
| --- | --- | --- | --- | --- | --- |
| Dihydrolipoyl dehydrogenase | *lpdA* | *E. coli* | Pyridine nucleotide-disulphide oxidoreductase, FAD/NAD(P)-binding domain (IPR023753) | Pyridine nucleotide-disulphide oxidoreductase (PF07992) | Pyridine nucleotide-disulphide oxidoreductases class-I active site |
| Alkyl hydroperoxide reductase | *ahpF* | *E. coli* | Pyridine nucleotide-disulphide oxidoreductase, FAD/NAD(P)-binding domain (IPR023753) | Pyridine nucleotide-disulphide oxidoreductase (PF07992) | Pyridine nucleotide-disulphide oxidoreductases class-II active site |
| NADH dehydrogenase | *ndh* | *E. coli* | Pyridine nucleotide-disulphide oxidoreductase, FAD/NAD(P)-binding domain (IPR023753) | Pyridine nucleotide-disulphide oxidoreductase (PF07992) | **-** |
| NADP^+^-specific glutamate dehydrogenase | *gdhA* | *E. coli* | NAD(P)-binding domain (IPR016040) | Glutamate/Leucine/Phenylalanine/Valine dehydrogenase (PF00208) | Glu / Leu / Phe / Val dehydrogenases active site |
| NADH dehydrogenase | *nuoF* | *E. coli* | NADH-ubiquinone oxidoreductase 51 kDa subunit, FMN-binding domain (IPR011538) | Respiratory-chain NADH dehydrogenase 51 Kd subunit (PF01512) | Respiratory-chain NADH dehydrogenase 51 Kd subunit signature 1 |
| Isocitrate dehydrogenase | *icd* | *E. coli* | Isopropylmalate dehydrogenase-like domain(IPR024084) | Isocitrate/isopropylmalate dehydrogenase (PF00180) | Isocitrate and isopropylmalate dehydrogenases signature |
| Nitrite reductase | *nirD* | *E. coli* | Rieske [2Fe-2S] iron-sulphur domain(IPR017941 | Rieske-like [2Fe-2S] domain (PF13806) | NADH-nitrite reductase subunit D family profile |
| Periplasmic nitrate reductase | *napA* | *E. coli* | Molybdopterin dinucleotide-binding domain(IPR006657) | Molybdopterin oxidoreductase (PF00384) | Prokaryotic molybdopterin oxidoreductases 4Fe-4S domain profile |
| Respiratory nitrate reductase | *narG* | *E. coli* | Molybdopterin oxidoreductase, 4Fe-4S domain(IPR006963) | Molybdopterin oxidoreductase (PF00384) | Prokaryotic molybdopterin oxidoreductases 4Fe-4S domain profile |
| Periplasmic nitrate reductase | *napA* | *Rhodobacter sphaeroides* | Molybdopterin oxidoreductase, 4Fe-4S domain(IPR006963) | Molybdopterin oxidoreductase (PF00384) | Prokaryotic molybdopterin oxidoreductases 4Fe-4S domain profile |
| Nitrous-oxide reductase | *nosZ* | *Paracoccus denitrificans* | Nitrous oxide reductase, N-terminal (IPR011045) | Cytochrome C oxidase subunit II, periplasmic domain (PF00116) | Cytochrome oxidase subunit II copper A binding domain profile |
| Catalase | *katA* | *Staphylococcus epidermidis* | Catalase-like domain (IPR020835) | Catalase (PF00199) | catalase family profile |
| Catalase-peroxidase | *katG* | *E. coli* | Haem peroxidase, plant/fungal/bacterial(IPR002016) | Peroxidase (PF00141) | Plant heme peroxidase family profile |
| Cytochrome d terminal oxidase | *cydA* | *E. coli* | - | Bacterial cytochrome ubiquinol oxidase (PF01654) | - |
| Nitrate reductase | *napA* | *P. pantotrophus* | Molybdopterin oxidoreductase, 4Fe-4S domain (IPR006963) | Molybdopterin oxidoreductase Fe4S4 domain | Prokaryotic molybdopterin oxidoreductases 4Fe-4S domain profile |
| dihydrolipoamide dehydrogenase | *lpdA* | *Aeromonas caviae* ST | Pyridine nucleotide-disulphide oxidoreductase, FAD/NAD(P)-binding domain (IPR023753) | Pyridine nucleotide-disulphide oxidoreductase | Pyridine nucleotide-disulphide oxidoreductases class-I active site |
| 6-phosphogluconate dehydrogenase | *gnd* | *E. coli* | NAD(P)-binding domain (IPR016040) | NAD binding domain of 6-phosphogluconate dehydrogenase | 6-phosphogluconate dehydrogenase signature |
| glutathione reductase | *gor* | *Pseudomonas* sp. BNF22 | Pyridine nucleotide-disulphide oxidoreductase, FAD/NAD(P)-binding domain (IPR023753) | Pyridine nucleotide-disulphide oxidoreductase | Pyridine nucleotide-disulphide oxidoreductases class-I active site |
| thioredoxin reductase | *trxB* | *E. coli* | Pyridine nucleotide-disulphide oxidoreductase, FAD/NAD(P)-binding domain (IPR023753) | Pyridine nucleotide-disulphide oxidoreductase | Pyridine nucleotide-disulphide oxidoreductases class-II active site |
| glutamate synthetase (GltD) | *murD* | *E. coli* | NAD(P)-binding domain (IPR016040) | Mur ligase middle domain | Prokaryotic membrane lipoprotein lipid attachment site profile |
| putative oxidoreductase | *ykgC* | *E. coli* | Pyridine nucleotide-disulphide oxidoreductase, FAD/NAD(P)-binding domain (IPR023753) | Pyridine nucleotide-disulphide oxidoreductase | Pyridine nucleotide-disulphide oxidoreductases class-I active site |
| glutathione reductase | *gorA* | *E. coli* | Pyridine nucleotide-disulphide oxidoreductase, FAD/NAD(P)-binding domain (IPR023753) | Pyridine nucleotide-disulphide oxidoreductase | Pyridine nucleotide-disulphide oxidoreductases class-I active site |
| nitrite reductase | *nirB* | *E. coli* | Pyridine nucleotide-disulphide oxidoreductase, FAD/NAD(P)-binding domain (IPR023753) | Pyridine nucleotide-disulphide oxidoreductase | Nitrite and sulfite reductases iron-sulfur/siroheme-binding site: |
| flavorubredoxin reductase | *norW* | *E. coli* | Pyridine nucleotide-disulphide oxidoreductase, FAD/NAD(P)-binding domain (IPR023753) | Pyridine nucleotide-disulphide oxidoreductase | - |
| mercuric reductase | *merA* | *PTp6 plasmid* | Pyridine nucleotide-disulphide oxidoreductase, FAD/NAD(P)-binding domain (IPR023753) | Pyridine nucleotide-disulphide oxidoreductase | Pyridine nucleotide-disulphide oxidoreductases class-I active site |
| pyridine nucleotide transhydrogenase | *SthA* | *E. coli* | FAD/NAD(P)-binding domain (IPR023753); Pyridine nucleotide-disulphide oxidoreductase (IPR004099) | - No hit - | Pyridine nucleotide-disulphide oxidoreductase |
| NADH-dependent dihydropyrimidine dehydrogenase subunit | *PreT* | *E. coli* | Dihydroprymidine dehydrogenase domain II (IPR028261); FAD/NAD(P)-binding domain (IPR023753) | - No hit - | Pyridine nucleotide-disulphide oxidoreductase |
| NADH-dependent dihydropyrimidine dehydrogenase subunit | *PreA* | *E. coli* | Fe-4S ferredoxin-type, iron-sulphur binding domain (IPR0178964) | 4FE4S_FER_2   4Fe-4S ferredoxin-type iron-sulfur binding domain profile | 4Fe-4S dicluster domain |
| 3-phenylpropionate dioxygenase, predicted ferredoxin reductase subunit | *HcaD* | *E. coli* | FAD/NAD(P)-binding domain (IPR023753); Pyridine nucleotide-disulphide oxidoreductase (IPR004099) | - No hit - | Pyridine nucleotide-disulphide oxidoreductase |
| UDP-galactopyranose mutase | *Glf* | *E. coli* | NAD(P)-binding domain (IPR016040); UDP-galactopyranose mutase (IPR015899) | - No hit - | NAD(P)-binding Rossmann-like domain |

Highlighted enzymes were characterized in this work.
