# Supplementary material for: Flavoprotein-Mediated Tellurite Reduction: Structural Basis and Applications to the Synthesis of Tellurium-Containing Nanostructures
Source: Front Microbiol. 2016 Jul 26;7:1160. doi: 10.3389/fmicb.2016.01160 (PMC4960239; doi:10.3389/fmicb.2016.01160)
Supplement: Supplementary file 3 [file Table_3.DOCX]

Supplementary Material

**Flavoprotein-mediated tellurite reduction: structural basis and applications to the synthesis oftellurium-containing nanostructures**

Mauricio Arenas-Salinas, Joaquín Vargas-Pérez, Wladimir Morales, Camilo Pinto, Pablo Muñoz, Fabián Cornejo, Benoit Pugin, Juan Sandoval, Waldo Díaz-Vásquez, Claudia Muñoz-Villagrán, Fernanda Rodríguez-Rojas, Eduardo Morales, Claudio C. Vásquez, FelipeArenas

**Correspondence to:** Felipe A. Arenas and/or Claudio C. Vásquez.E-mails: [felipe.arenass@usach.cl](mailto:felipe.arenass@usach.cl); [claudio.vasquez@usach.cl](mailto:claudio.vasquez@usach.cl)

**Table 3S.** Structure comparisons

|  | Protein 1 | PDB_ID | Protein 2 | PDB_ID | % ID | SSAP Score (1 – 100) | RMSD (Å) |
| --- | --- | --- | --- | --- | --- | --- | --- |
| 1 | TrxB | 1CL0 | E3 | 4JDR | 14 | 71.5 | 5.29 |
| 2 | TrxB | 1CL0 | GorA | 1GER | 20 | 73.77 | 6.73 |
| **3** | **TrxB** | **1CL0** | **AhpF** | **1FL2** | **33** | **89.05** | **1.55** |
| 4 | AhpF | 1FL2 | E3 | 4JDR | 15 | 72.02 | 6.32 |
| 5 | AhpF | 1FL2 | GorA | 1GER | 17 | 73.23 | 6.59 |
| 6 | **GorA** | **1GER** | **E3** | **4JDR** | **25** | **86.08** | **2.04** |
| 7 | YkgC | - | TrxB | 1CL0 | 15 | 72.83 | 4.48 |
| 8 | **YkgC** | **-** | **GorA** | **1GER** | **26** | **87.23** | **2.05** |
| 9 | **YkgC** | **-** | **E3** | **4JDR** | **27** | **89.67** | **1.09** |
| 10 | YkgC | - | AhpF | 1FL2 | 15 | 73.5 | 5.62 |

Comparisons were carried out according to STAMP (Russell and Barton, 1992) and SSAP methods (Orengo and Taylor, 1996). Comparison between enzymes of the same class are shown in bold.
