# Supplementary material for: Flavoprotein-Mediated Tellurite Reduction: Structural Basis and Applications to the Synthesis of Tellurium-Containing Nanostructures
Source: Front Microbiol. 2016 Jul 26;7:1160. doi: 10.3389/fmicb.2016.01160 (PMC4960239; doi:10.3389/fmicb.2016.01160)
Supplement: Supplementary file 4 [file Table_4.DOCX]

Supplementary Material

**Flavoprotein-mediated tellurite reduction: structural basis and applications to the synthesis of tellurium-containing nanostructures**

Mauricio Arenas-Salinas, Joaquín Vargas-Pérez, Wladimir Morales, Camilo Pinto, Pablo Muñoz, Fabián Cornejo, Benoit Pugin, Juan Sandoval, Waldo Díaz-Vásquez, Claudia Muñoz-Villagrán, Fernanda Rodríguez-Rojas, Eduardo Morales, Claudio C. Vásquez, Felipe Arenas

**Correspondence to:** Felipe A. Arenas and/or Claudio C. Vásquez. E-mails: [felipe.arenass@usach.cl](mailto:felipe.arenass@usach.cl); [claudio.vasquez@usach.cl](mailto:claudio.vasquez@usach.cl)

**Table 4S. Distances between relevant atoms involved in the TR activity of *E. coli* flavoproteins**

| Enzyme | PDB | Distance 1  Cα-Cα | Distance 2  CYS1 SH-FAD | Distance 3  CYS2 SH-FAD | Distance 4  CYS1 SH-CYS2SH | Distance 5  CYS2-N5FAD |
| --- | --- | --- | --- | --- | --- | --- |
| TrxB | 1CL0 | 5.27  CYS135C:CYS138CA | 3.40  CYS138S:FAD500C4 | 6.30  CYS135S:FAD500C4 | 3.09  CYS135S:CYS138S | 6.62  CYS135S:N5FAD |
| AhpF | 1FL2 | 5.27  CYS345C:CYS348CA | 3.07  CYS348S:FAD522C4 | 4.92  CYS345S:FAD522C4 | 2.02  CYS345S:CYS348S | 4.88  CYS345S:N5FAD |
| GorA | 1GER | 4.59  CYS42CA:CYS47CA | 3.48  CYS47S:FAD451C4 | 5.45  CYS42S:FAD451C4 | 2.05  CYS42S:CYS47S | 6.04  CYS42S:N5FAD |
| E3 | 4JDR | 4.85  CYS45CA:CYS50CA | 4.61  CYS50S:FAD501C4 | 6.04  CYS45S:FAD501C4 | 1.96  CYS45S:CYS50S | 6.35  CYS45S:N5FAD |

Distances (Å) were calculated using the software VMD
