# Supplementary material for: Flavoprotein-Mediated Tellurite Reduction: Structural Basis and Applications to the Synthesis of Tellurium-Containing Nanostructures
Source: Front Microbiol. 2016 Jul 26;7:1160. doi: 10.3389/fmicb.2016.01160 (PMC4960239; doi:10.3389/fmicb.2016.01160)
Supplement: Supplementary file 5 [file Table_5.DOCX]

Supplementary Material

**Flavoprotein-mediated tellurite reduction: structural basis and applications to the synthesis oftellurium-containing nanostructures**

Mauricio Arenas-Salinas, Joaquín Vargas-Pérez, Wladimir Morales, Camilo Pinto, Pablo Muñoz, Fabián Cornejo, Benoit Pugin, Juan Sandoval, Waldo Díaz-Vásquez, Claudia Muñoz-Villagrán, Fernanda Rodríguez-Rojas, Eduardo Morales, Claudio C. Vásquez, FelipeArenas

**Correspondence to:** Felipe A. Arenas and/or Claudio C. Vásquez.E-mails: [felipe.arenass@usach.cl](mailto:felipe.arenass@usach.cl); [claudio.vasquez@usach.cl](mailto:claudio.vasquez@usach.cl)

**Table 5S. Biological flavoprotein-catalyzed reactions**

| **Enzyme** | Catalyzed reaction | **Preference cofactor** |
| --- | --- | --- |
| GorA | glutathione disulfide + NADPH + H^+^ → 2 glutathione + NADP^+^ | NADPH |
| E3 | pyruvate + coenzyme A + NAD^+^ → acetyl-CoA + CO_2_ + NADH (catalyzed by complex) | NADH |
| TrxB | [oxidizedthioredoxin + NADPH + H^+^](http://www.ecocyc.org/ECOLI/NEW-IMAGE?type=REACTION&object=THIOREDOXIN-REDUCT-NADPH-RXN)→ reduced thioredoxin + NADP^+^ | NADPH |
| AhpF | organic hydroperoxide + NADH + H^+^ → alcohol + NAD^+^ + H_2_O (catalyzed by complex) | NADH |
| YkgC | Not known | - |
| NorW | [oxidizedflavorubredoxin + NADH →reducedflavorubredoxin + NAD^+^](http://www.ecocyc.org/ECOLI/NEW-IMAGE?type=REACTION&object=RXN0-7108) | NADH |
| SthA | NADPH + NAD^+^ → NADH + NADP^+^ | NADPH |
| PreT | 5,6-dihydrouracil + NAD^+^ ↔ uracil + NADH + H^+^ (catalyzedbycomplex) | NADH |
| PreA | 5,6-dihydrouracil + NAD^+^ ↔ uracil + NADH + H^+^ (catalyzedbycomplex) | - |
| HcaD | trans-cinnamate + NADH + oxygen + H^+^ → (2E)-3-(5,6-dihydroxycyclohexa-1,3-dien-1-yl)prop-2-enoate + NAD^+^ (catalyzedbycomplex) | NADH |
| Glf | UDP-α-D-galactose ↔ UDP-α-D-galactofuranose | - |
| NirB | nitrite + 3 NADH + 5 H^+^ → ammonium + 3 NAD^+^ + 2 H2O | NADH |
| GltD | L-glutamine + 2-oxoglutarate + NADPH + H^+^→2 L-glutamate + NADP^+^(catalyzedbycomplex) | NADPH |
