# Supplementary figures and images for: Flavoprotein-Mediated Tellurite Reduction: Structural Basis and Applications to the Synthesis of Tellurium-Containing Nanostructures
Source: Front Microbiol. 2016 Jul 26;7:1160. doi: 10.3389/fmicb.2016.01160 (PMC4960239; doi:10.3389/fmicb.2016.01160)

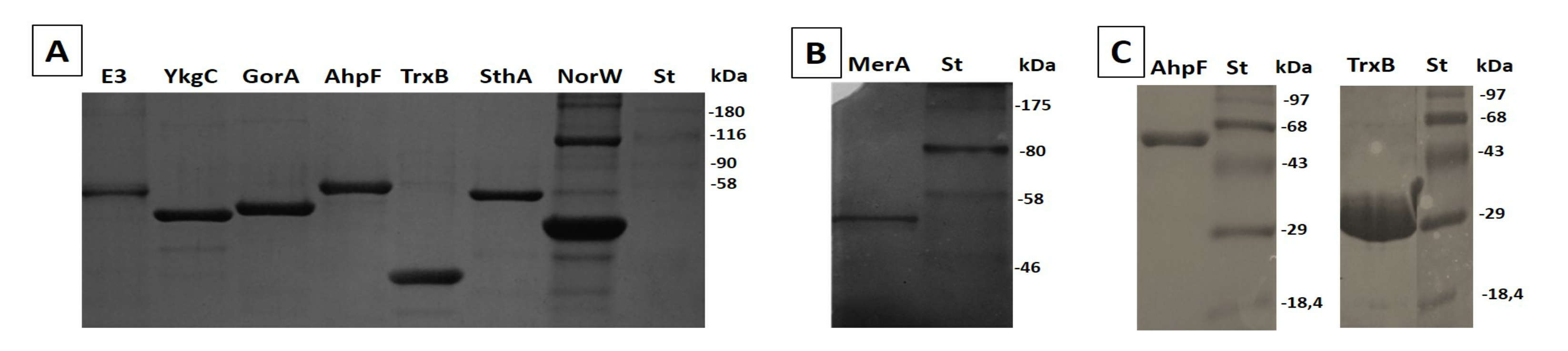

Supplement: FIGURE S1 — SDS-PAGE of purified putative tellurite reductases. (A) Purified E. coli flavoproteins E3 (∼56 kDa), YkgC (∼48 kDa), GorA (∼49 kDa), AhpF (∼56 kDa), TrxB (∼35 kDa), SthA (∼52 kDa), and NorW (∼41 kDa) were visualized by PAGE-SDS. Each lane contained 15 μg of protein. (B) Mercuric reductase (MerA, ∼56 kDa, 10 μg) from the environmental plasmid pTP6. (C) S. haemolyticus BNF01 flavoproteins AhpF (∼60 kDa, 10 μg) and TrxB (∼35 kDa, 20 μg). Protein standards (St) were SDS7B2 Sigma (A), Broad Range NEB (B) and MW High-range Gibco BRL. [file Image_1.TIF]

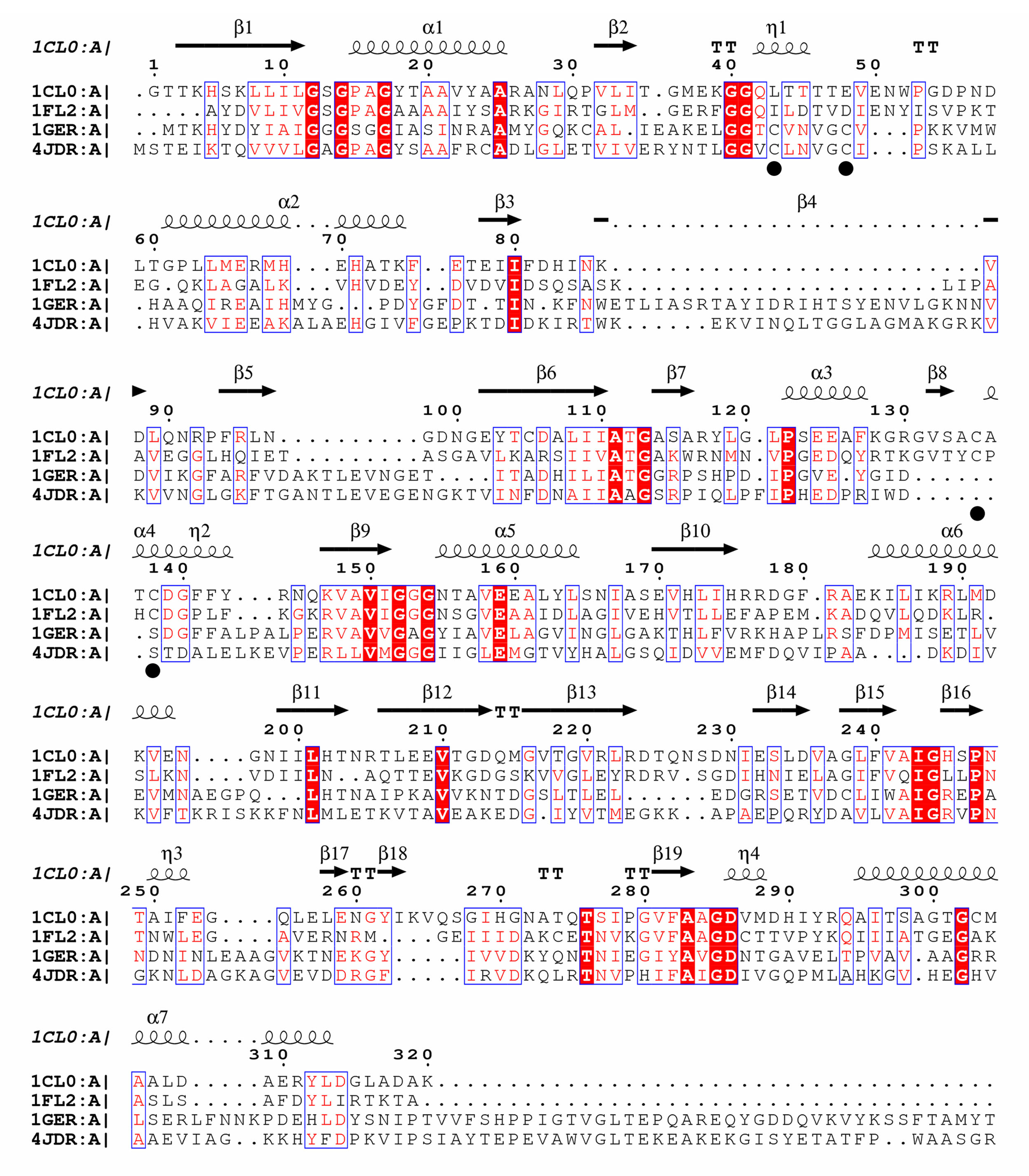

Supplement: FIGURE S2 — Amino acid sequence alignment of the indicated E. coli flavoproteins. Secondary structure elements of E3 (PDB_ID: 4JDR), GorA (PDB_ID: 1GER), AhpF (PDB_ID: 1FL2), and TrxB (PDB_ID: 1CL0) are indicated at the top (hairsprings: α-helix; arrows, β-sheets). Cys residues are indicated by black dots. [file Image_2.TIF]

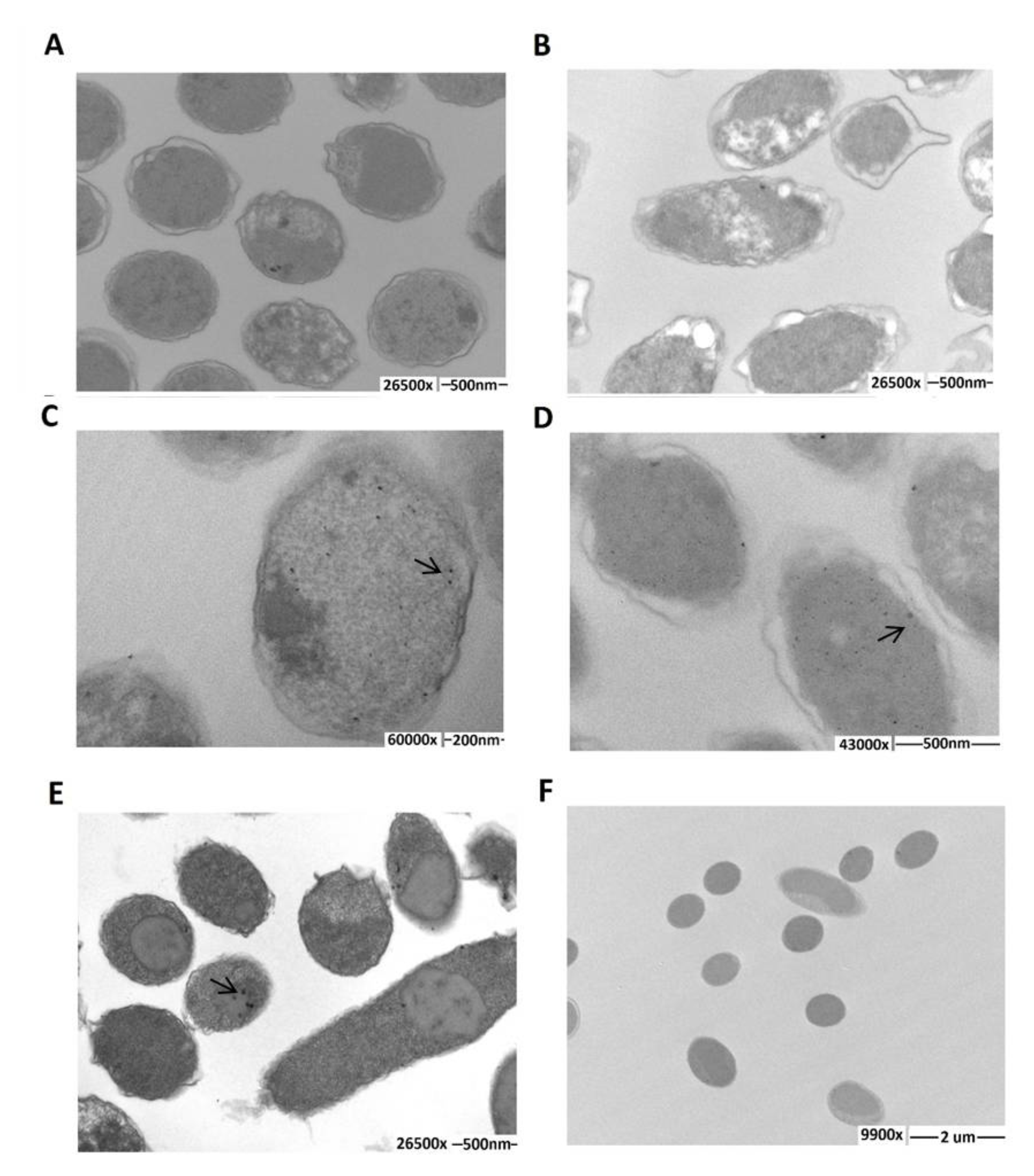

Supplement: FIGURE S3 — Transmission electron microscopy of in vivo synthesized electron-dense assemblies. The structures resulting from the in vivo tellurite reduction by E. coli strains overproducing AhpF (A), E3 (B), GorA (C), YkgC (D), TrxB (E), and NorW (F) were visualized by TEM as described in Section “Materials and Methods.” [file Image_3.TIF]
